# Recommendations for the development and use of technology to support people living with dementia and caregivers: A Delphi study

**DOI:** 10.1002/alz.70755

**Published:** 2025-09-29

**Authors:** Duygu Sezgin, Flora‐Marie Hegerath‐Segler, Hannah Christie, Jackie Poos, Kevin Cullen, Emer Meagher, Manuel Gonçalves‐Pereira, Horst Christian Vollmar, Cíara O'Reilly, Aisling Mitchell, Salman Alabdulkder, David Neal, Sarah Janus

**Affiliations:** ^1^ School of Nursing and Midwifery University of Galway Galway City Galway Ireland; ^2^ Institute of General Practice and Family Medicine (AM RUB) Medical Faculty Ruhr University Bochum Universitätsstraße 150 Bochum Germany; ^3^ School of Population Health RCSI University of Medicine and Health Sciences Dublin Ireland; ^4^ Alzheimer Centre and Department of Neurology Erasmus MC University Medical Centre Rotterdam The Netherlands; ^5^ Dementia Research Advisory Team The Alzheimer Society of Ireland Dublin Ireland; ^6^ NOVA Medical School, Faculdade de Ciências Médicas Universidade Nova de Lisboa; CHRC, Comprehensive Health Research Center, LA‐REAL Lisbon Portugal; ^7^ Research Project Office The Alzheimer Society of Ireland Dublin Ireland; ^8^ Department of Medical Informatics eHealth Living & Learning Lab Amsterdam UMC Amsterdam The Netherlands; ^9^ Department Primary‐ and Long‐term Care University Medical Centre Groningen Groningen The Netherlands

**Keywords:** assistive technology, caregivers, Delphi study, dementia, people living with dementia, technology development

## Abstract

**INTRODUCTION:**

This Delphi study, conducted by the INTERDEM Assistive Technology (AT) taskforce, explores existing and future challenges in the development, usability, cost‐effectiveness, implementation, and ethics of AT for people living with dementia and caregivers. The study aims to identify key priorities and actions to address these challenges.

**METHODS:**

A two‐round modified electronic Delphi study was conducted with experts from health and social care, dementia research, technology development, people living with dementia, and caregivers.

**RESULTS:**

Consensus was reached on 23 key statements highlighting the need for a user‐centered approach to AT development. Priorities included integrating AT into care plans, enhancing accessibility, and ensuring collaboration between stakeholders. Ethical considerations, digital literacy, and equitable access were also emphasized.

**DISCUSSION:**

Our findings refine and update previous recommendations on AT development and use. This Delphi study contributes to guiding future research, policy, and practice to ensure AT effectively supports people living with dementia and caregivers.

**Highlights:**

- Co‐designing technologies with users is crucial to ensure relevance and usability.
- Priorities in developing technology include improving access and affordability.
- Technology development should aim reducing disparities in digital access.
- Future research on technology should be inclusive and reflect real‐life needs.

## 1 INTRODUCTION

Dementia is a growing global public health concern, placing significant strain on individuals, families, and healthcare systems.^1^ As societies age, the need for effective post‐diagnostic support grows, to help people living with dementia maintain their autonomy and enhance their quality of life, well‐being, and sense of normality.^2^ Assistive technologies (ATs) may play a crucial role in achieving these goals. By supporting day‐to‐day living, preserving dignity, fostering social connection, and enabling meaningful engagement in daily activities,^3^, ^4^ ATs offer valuable tools for people living with dementia and caregivers.

Assistive technologies include a wide range of electronic devices and systems, from electronic calendars and web‐based information services to video‐calling and activity support tools.^5^ Defined by the World Health Organization^6^ as any product primarily designed to maintain or improve an individual's functioning and independence, ATs are increasingly recognized as adaptable and cost‐effective solutions to support memory, communication, psychological well‐being, and activities of daily living.^7^

Despite its potential, the adoption of ATs by people living with dementia, informal caregivers (henceforth ‘caregivers’), and healthcare professionals remains challenging. Research has identified several barriers and facilitators. Barriers include technology anxiety, limited perceived benefits, a mismatch between devices and needs, resistance to use ATs, negative attitudes, lack of knowledge, inaccessibility, poor design, and ethical issues.^3^, ^8^, ^9^ Cognitive deterioration can pose significant challenges to the adoption and continued use of technology for people living with dementia as difficulties in learning and remembering how to use ATs are a reality due to cognitive impairments.^10^ Caregivers and healthcare professionals also express concerns about depersonalization, job displacement, and increased social isolation resulting from ATs integration in dementia care. The facilitators of ATs include perceived benefit, affordability and accessibility, personalization, adaptability, and integration into practice.^3^, ^4^, ^8^, ^10^ Technology use can risk reducing patients to mere bodies rather than whole individuals, contradicting the holistic approach central to care. However, replacing personal care with technology may undervalue caregiving skills and divert attention from staff training needs.^11^ Additionally, ethical concerns regarding autonomy, privacy, and data security further complicate its widespread adoption.^12^, ^13^

**RESEARCH IN CONTEXT**

1. **Systematic review**: We conducted a Delphi study involving experts from the perspectives of people with dementia, caregivers, care professionals, technology developers. Using two‐round online Delphi surveys, we aimed to identify challenges with development and use of assistive technologies for people living with dementia and caregivers and recommendations to address them. This study facilitated representation of multi‐stakeholders.
2. **Interpretation**: Our findings demonstrate that co‐designing assistive technologies with people living with dementia and caregivers is crucial for ensuring usability and relevance. Development efforts must priorities accessibility, and affordability, and reducing the digital divide.
3. **Future directions**: Future assistive technology research should be more inclusive, involving technology developers and diverse communities, and reflect real‐life experiences to enhance dementia care and support. Future studies on this topic could engage more international stakeholders from non—English‐speaking countries to increase their representation when further exploring assistive technology development and use in dementia care.

The INTERDEM network, a pan‐European consortium of over 250 researchers from 19 countries,^14^ has highlighted the need for ATs that effectively supports people with dementia and caregivers. Notwithstanding, the 2017 position paper^15^ discussed the key challenges according to the following domains: usability, development, ethics, cost‐effectiveness, and implementation across three application areas (daily life management, engagement in meaningful activities, and dementia care provision). The paper reviewed existing technologies and research gaps in ATs for dementia. Key challenges in design, accessibility, and integration highlight the need for clear development priorities, especially as this is a rapidly evolving field.^16^ The current study aims to identify these priorities using expert consensus. This approach helps ensure that ATs are user‐friendly and aligned with end‐user needs, which is essential for optimizing their place in dementia care.

This Delphi study was conducted by the INTERDEM AT taskforce members to explore the existing and future challenges to development, usability, cost‐effectiveness, implementation, and ethics concerning AT use by people living with dementia and caregivers. It also aims to identify key priorities and actions to address challenges in ATs for dementia. Unlike previous studies focusing mainly on researchers, this study adopts a multi‐stakeholder approach, involving people living with dementia, caregivers, care professionals, and technology developers. By integrating diverse perspectives, it seeks to better understand barriers to ATs adoption and develop practical solutions. The findings will inform priorities and actions to address challenges for future design and implementation of ATs in dementia care. Therefore, it explores the research questions below:

1. According to the researchers, practitioners, and people living with dementia and caregivers, what are the most important challenges to development, usability, cost‐effectiveness, implementation and ethics concerning ATs for people living with dementia and caregivers?

2. Which actions do researchers, practitioners, and people living with dementia and caregivers believe should be prioritized to address these challenges?

## 2 METHODS

### 2.1 Design

This two‐round modified electronic Delphi study involved experts representing perspectives from health and social care services, dementia research, technology developers for dementia care, and people living with dementia and caregivers. The Delphi method was chosen to systematically collate and analyze the views of the experts in dementia care. To ensure the lived experience of dementia was incorporated throughout the research process, Patient and Public Involvement (PPI) contributors were sourced from The Alzheimer Society of Ireland's dedicated PPI panel – the Dementia Research Advisory Team. The PPI Contributors were integral members of the independent research team, providing valuable insights from the initial conceptualization stage and continuing to inform and shape the study throughout its duration. Guidance on Conducting and Reporting Delphi Studies (CREDES) was followed for conducting the study and reporting process.^17^

### 2.2 Participants and recruitment

All members of the INTERDEM group and people in their networks including researchers, practitioners, and people living with dementia and caregivers were invited to take part. However, aiming at a broad range of expertise among the participants, INTERDEM Academy members were also invited to take part in the study. The INTERDEM Academy is part of INTERDEM, established to promote the career development and capacity building of dementia care researchers.^14^ Representatives from INTERDEM and INTERDEM Academy acted as gatekeepers for this study and sent an invitation email to all their members, who were asked to share the study link within their networks.

In efforts to achieve and further enhance the representation of people living with dementia and caregivers as well as care practitioners, the study invitation was also sent to the Alzheimer Europe PPI steering group, and national dementia care and support groups known to the research team such as the Alzheimer Portugal associates, Dementia National Coordination teams, German Alzheimer Society, Dutch Memory Clinic Network, and to the relevant members of The Alzheimer Society of Ireland's national research participant recruitment service, TeamUp for Dementia Research. Representatives and contact persons from these organizations were also asked to act as gatekeepers and invite their members to take part in the study. Due to this broad recruitment strategy, the total target sample size is unknown, but it is estimated that at least 350 individuals were invited for the first Delphi survey round.

Inclusion criteria were (1) being a member of the INTERDEM or INTERDEM Academy and being on their mailing list *or* being in the network of an INTERDEM or INTERDEM Academy member as a researcher, practitioner, or person living with dementia or caregiver, *or* (for those identified via other professional or patient and family support organization) being a person living with dementia or caregiver or a practitioner who delivers care to people with dementia; and (2) agreeing to take part in the study by giving informed consent before the first Delphi survey round. Those who did not meet the inclusion criteria above were excluded.

### 2.3 Data collection

**FIGURE 1:**
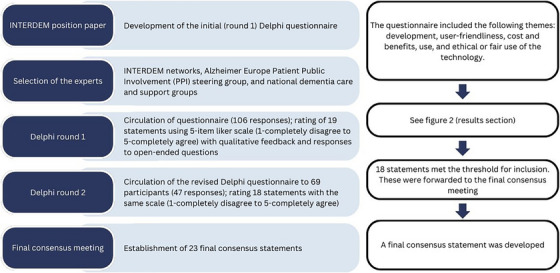
Flow chart of methods.

Data were collected through two rounds of online Delphi surveys. Two‐round Delphi is a common approach and, in some cases, consensus can be established even after the first round.^17^, ^18^ In this study, the second round was deemed necessary to address the input provided by the participants in the first round. QuestionPro online surveying tool^19^ was used for data collection. Figure 1 presents a summary of the data collection methods and process.

#### 2.3.1 Round 1

The round 1 survey questions (Appendix A) were drafted based on a previous position paper published by the INTERDEM Assistive Technology taskforce members^15^ and they were further refined following the PPI contributors’ review and feedback. There were 19 statements under five main sections for circulation in round 1. These sections were related to the existing and future challenges regarding development, usability, cost‐effectiveness, implementation, and ethics concerning assistive technology for a person living with dementia and caregivers. The wording of these section headings and statements were simplified following the PPI contributors’ review, which emphasized the importance of using plain language in research involving people living with dementia. The simplified version of the headings was as follows: development of technology, user‐friendliness of the technology, costs and benefits of the technology, use of technology, and ethical or fair use of technology.

All participants were asked to rate the statements using a five‐item Likert scale between the options of (1) strongly disagree and (5) strongly agree or indicate “I don't have an opinion”. Under the statements, they were given the opportunity to add open‐text comments related to their suggestions for developing and using technology to support people living with dementia and caregivers, challenges they observed and their specific recommendations to address these challenges. The survey also had questions about demographic and background information such as age, gender, geographical location, years of experience living with or working in the field of dementia, use of assistive technology in daily life, and the interest group they represent, that is, person living with dementia, caregiver, health or social care professional, researcher, technology developer, other. The content and formatting of round 1 were piloted within the study team before the data collection commenced and revised accordingly. The first round of the Delphi survey was sent out on the 27th of September 2024 and closed on the 29th of October 2024.

#### 2.3.2 Round 2

Participants of round 1 were asked to provide their email addresses if they were interested in taking part in round 2. Following round 1, participants were provided with a summary of the results. Any changes made to the statements circulated in round 2 based on the open‐text comments were highlighted and the rationale for these changes was provided. Similar to the first round, the second round included questions about demographic and background information, followed by 18 statements under the same five main sections. Same as the first round, statements were rated (1) strongly disagree to (5) strongly agree, with “I don't have an opinion” being an option. There was an overall feedback and optional open‐text comment section at the end of the survey. This round 2 survey was sent out on November 19, 2024 and closed on December 12, 2024. In the meantime, weekly reminders were sent to encourage participation.

### 2.4 Data analysis

#### 2.4.1 Round 1

Once the responses were collected, they were analyzed using quantitative (descriptive statistics such as frequencies and mean) and qualitative (inductive thematic analysis) methods. The qualitative and quantitative analysis results were combined and used to amend the existing statements or devise new statements based on the open‐text comments provided by the participants. The revised statements were sent out to the participants in round 2. The criteria for establishing consensus were based on statements rated “4‐ agree” or “5‐ strongly agree” by 75% or over and rated “1‐ strongly disagree” or “2‐ disagree” by less than 15% of the participants.^18^ The establishment of consensus by percentage agreement is the most common approach in Delphi studies as highlighted in a systematic review of 329 Delphi studies.^20^ Statements that did not reach consensus were revised based on the open‐text comments and sent out for review in round 2.

#### 2.4.2 Round 2

The data analysis of round 2 was only based on quantitative data analysis of the responses to the statements. The same consensus criteria were applied to the round 2 data. The level of agreement and disagreement between the rounds was identified based on comparisons of percentage ratings for each statement.

### 2.5 Ethics and data protection

The ethics approval for this study was granted by the University of Galway Research Ethics Committee (reference number: 2024.06.025). The researchers followed the General Data Protection Regulations (2016) for data collection, processing, and reporting. All participants gave informed consent before responding to each Delphi round.

## 3 RESULTS

Characteristics of the participants for round 1 and 2 are presented in Table 1. In the first round, 19 statements were evaluated and refined based on the participant feedback. In the second round, 13 revised and 5 new statements were re‐evaluated, all of which achieved consensus.

**TABLE 1:** Participant characteristics in rounds 1 and 2.

Characteristics	Round 1 (*n* = 106) *n* (%)	Round 2 (*n* = 47) *n* (%)
Age (years)		
18–34	18 (16.98)	8 (17.02)
35–44	15 (14.15)	7 (14.89)
45–54	23 (21.70)	11 (23.40)
55–64	26 (24.53)	11 (23.40)
65–74	20 (18.87)	8 (17.02)
75 and over	4 (3.77)	2 (4.26)
Gender		
Male	29 (27.36)	15 (31.91)
Female	76 (71.70)	32 (68.09)
Non‐binary	1 (0.94)	0 (0)
Interest group (can select multiple)		
Person living with dementia	15 (12.00)	10 (16.95)
Caregiver	27 (21.60)	10 (16.95)
Practitioner, for example, health or social care professional	27 (21.60)	12 (20.34)
Researcher	47 (37.60)	22 (37.29)
Technology developers	2 (1.60)	1 (1.69)
Other	7 (5.60)	4 (6.78)
Years of experience working or living with dementia		
Less than 1	6 (5.66)	1 (2.13)
Between 1–3	18 (16.98)	9 (19.15)
Between 3–5	14 (13.21)	6 (12.77)
Between 5–10	25 (23.58)	9 (19.15)
More than 10	43 (40.57)	22 (46.81)

### 3.1 Round 1

**FIGURE 2:**
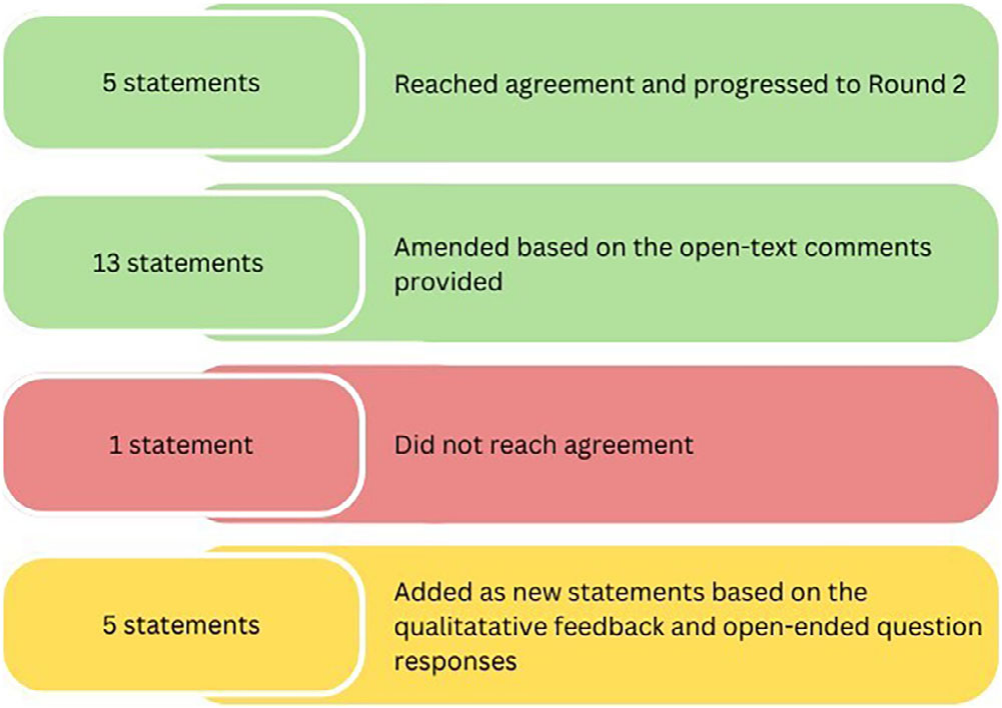
Round 1 Delphi results.

A total of 106 participants from 16 countries responded to the round 1 survey. Those were Ireland (*n* = 42); the United Kingdom (*n* = 25), including England (*n* = 8), Scotland (*n* = 2), Northern Ireland and Wales (*n* = 1 each), and not specified (*n* = 13); Portugal (*n* = 16), the Netherlands (*n* = 10), Belgium and Germany (*n* = 3 each), followed by Australia, Greece, Italy, Norway, Poland, Spain, and Switzerland (*n* = 1 each). Out of the 19 statements, 18 of them were agreed or strongly agreed by more than 75% of the participants. Only one statement did not reach the 75% consensus agreement threshold and was therefore excluded. This statement was *“Technology does not always fit well in the current healthcare practices and this is a barrier to its use.”* No open‐text comments were provided by the participants suggesting amendments to this statement. Of the remaining 18 statements, consensus was established for five, based on the 75% threshold, and no further contributions were found in the open‐text comments provided by the participants. The other 13 statements were amended based on participant comments provided in round 1 and re‐circulated in round 2. Lastly, five new statements were developed based on the participant comments and these were circulated in round 2 (Figure 2). A detailed breakdown of rounds 1 and 2 statement rankings is available in Appendix A. The process of mapping the open‐text comments to the statements, amending them accordingly, and developing new statements is presented in Appendix B.

#### 3.1.1 Summary of the open‐text comments based on the participants’ background

**FIGURE 3:**
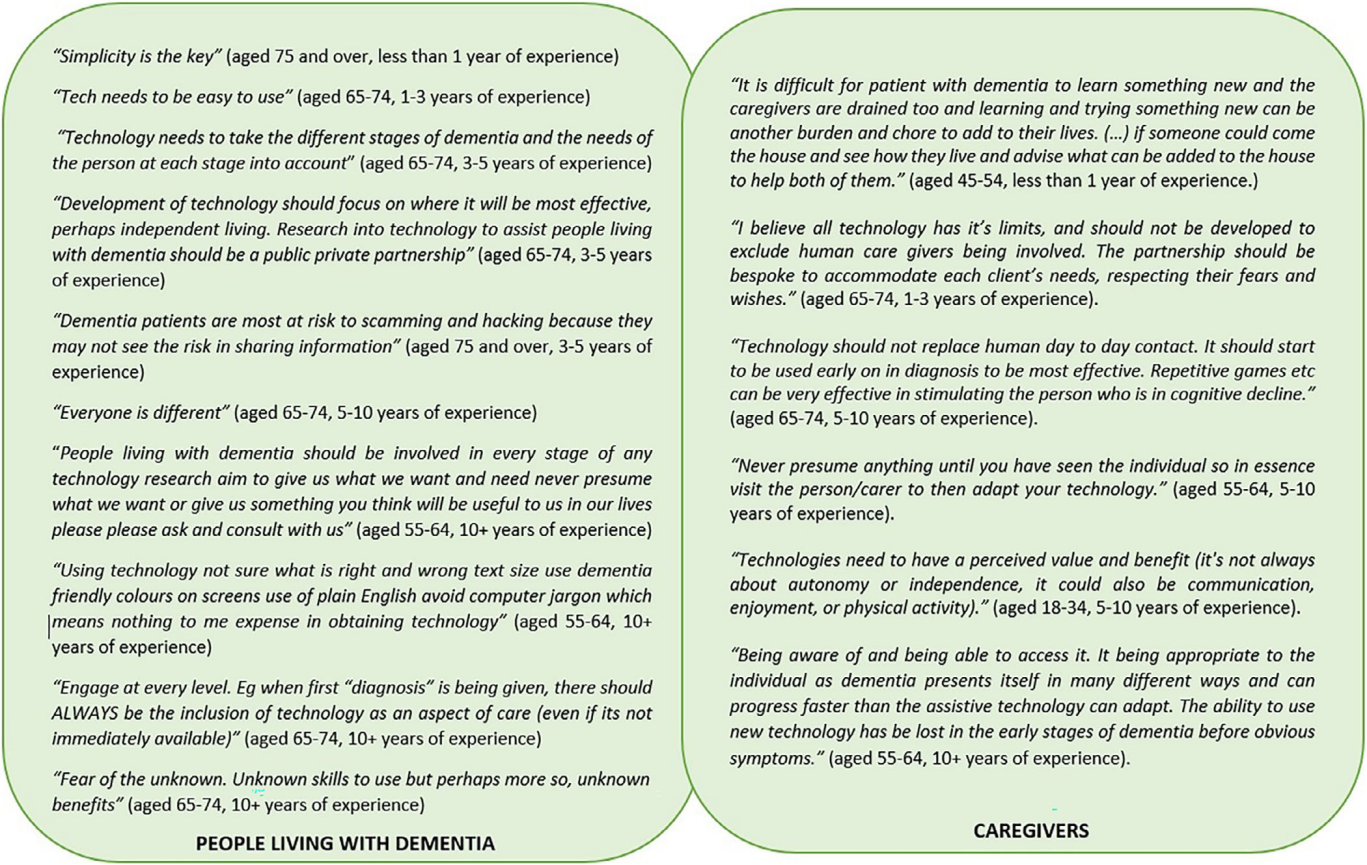
A selection of the comments provided by people living with dementia (box on the left) and caregivers (box on the right) with various level of experience.

In total, there were 68 comments related to suggestions, 82 about future challenges, and 73 comments on recommendations to address these challenges. These comments were analyzed to make comparisons based on the participants’ age, years of experience living with or working in the field of dementia, and the interest group they were representing, for example, person living with dementia, researcher, or practitioner. Figure 3 presents a selection of comments provided by people living with dementia and caregivers. People living with dementia, caregivers, and researchers highlighted that technology development should be an interdisciplinary and collaborative process. Some people living with dementia suggested the use of artificial intelligence (AI) as a communication tool. An example of this approach was the creation of an individual communication profile to enable them to express themselves as they wish, should their disease progresses to a point where they are no longer able to do so. Participants from almost all age groups (18–34, 45–54, 55–64, and 65–74 years) suggested that technology should be adaptable and reflective of the changing needs of people living with dementia at different stages of their journey. Furthermore, a number of participants, mostly those aged 65–74 years, suggested that technology should not replace human interactions or social contact. Regarding future challenges, people living with dementia, caregivers, and researchers identified cost‐effectiveness and digital illiteracy as key barriers to the use of technology. Participants from all age groups had concerns over the ethical or privacy issues that some new technologies may bring and mentioned that they could add to the carers’ responsibilities. As a suggestion to manage these challenges, the participants advocated for the inclusion of a wide range of stakeholders, but particularly people living with dementia and caregivers, in the development of technology. All age groups echoed this statement and advocated for the promotion of digital literacy among people living with dementia and caregivers.

Overall, the participants with less than a year of experience working or living with dementia mentioned the need for simple, user‐friendly technology that promotes independence while being easy to implement. Those with 1–3 years of experience emphasized the importance of ensuring that technology is dementia‐friendly, simple to use, and affordable. They also highlighted the need for more personalized support, including training for caregivers and dementia advisors, to help effectively incorporate technology. Grants and collaborations were proposed as strategies to address financial hurdles and improving technology availability and quality. Comments from those with 3–5 years of experience also emphasized the importance of developing simple, adaptable, and affordable technologies tailored the specific needs of people living with dementia and caregivers. Equity was highlighted as a major concern, as these technologies may become inaccessible to certain groups, particularly those in low‐income communities. The participants highlighted that addressing these issues will require both technological innovation and a dedication to ensuring that these solutions are inclusive, inexpensive for the individual, and respectful of the privacy and autonomy of those who use them. Responses received by those who had 5–10 years of experience emphasized several themes: personalization, collaboration, simplicity, and low costs are all essential for developing technologies that actually assist people living with dementia and caregivers. They noted an urgent need for education, privacy protection, and the proper integration of technology into established healthcare institutions. The most experienced group with over 10 years of experience highlighted a policy‐driven approach, emphasizing regulation, ethical pricing, and equitable access. They urged intensive family caregiver and healthcare professional training, as well as continued research into ATs' long‐term efficacy. Privacy problems, computer literacy, and the necessity for ongoing support are among the primary challenges they identified. Overall, simplicity, cost‐effectiveness, and affordability were highlighted by participants at all levels of experience who were based in different countries. Ethical concerns including privacy risks and inequalities in access and training needs were raised across those with various levels of experience.

### 3.2 Round 2

Sixty‐nine participants from round 1 expressed interest in taking part in round 2 and provided their contact information. Of these, 47 participants (68%) from 10 countries took part in the round 2 survey. They were from Ireland (*n* = 18), England (*n* = 9), Portugal and the Netherlands (*n* = 7 each), followed by Australia, Germany, Italy, Northern Ireland, Scotland, and Wales (*n* = 1 each).

In the round 2 survey, all of the 13 revised statements from round 1 and the 5 new statements reached the 75% consensus threshold. Further details of the round 2 ratings are available in Appendix A. Following the round 2 Delphi survey, consensus was reached on a total of 23 statements about the development of technology, user‐friendliness of the technology, benefits of the technology, use of technology, and ethical or fair use of technology to support people living with dementia and caregivers (Table 2).

**TABLE 2:** Consensus statement on the development and use of technology to support people living with dementia and caregivers.

Development of technology	Degree of consensus (% strongly agree or agree)
People living with dementia and family caregivers must be at the forefront at all stages of the design and development of technology to support them.	95.74%
Tools should be customizable to each specific stage of dementia to address the changing needs of people living with dementia and caregivers.	91.49%
Tools should be provided to people living with dementia as part of their care plan and incorporated in their daily care routines and at the appropriate time to their needs.	85.11%
Researchers and technology developers must learn about the needs of people living with dementia by including them in the research and design process.	97.87%
Tools should be designed to complement human interaction, not replace it.	95.74%
Collaboration across health and social care practitioners and researchers, technology developers, people living with dementia and caregivers is essential to create effective and accessible technologies	100%
User‐friendliness of the technology	
Research should find out the needs of people living with dementia and how to address them.	97.17%
Research is needed to find out how technology can help them live independently.	92.45%
Research is needed so people living with dementia can use technology for independent living, no matter their background or education.	87.74%
Researchers and developers must recognize that many people living with dementia and caregivers have limited experience with technology.	85.11%
It is essential to design and develop technology that is simple and accessible to meet their needs and enhance their quality of life.	97.87%
Costs and benefits of the technology	
Researchers and developers should make technology affordable for people living with dementia.	89.62%
Researchers should study if technology provides value for money.	80.19%
Dementia manifests itself differently for each person but everyone goes through similar stages. Technology needs to support the specific needs of people living with dementia at each stage of the disease.	91.49%
Researchers and developers should develop clear criteria to validate the effectiveness of technology to benefit people living with dementia and caregivers (such as helping them stay connected or manage day‐to‐day tasks).	91.49%
Technology should ease caregiver responsibilities, not increase them.	95.74%
Use of technology	
It is essential to provide education for people living with dementia and caregivers and train care staff about how to use the technology. A lack of information and knowledge is a barrier to the use of technology.	95.74%
It is important that experts are available to provide individual, personalized support during installation and continued use of the technology (such as dedicated end‐user support and providing training through community services).	95.74%
Researchers and developers should focus on who will use technology in everyday environments to ensure it meets user needs, integrates into routines and is compatible.	97.87%
Ethical or fair use of technology	
Technologies for people living with dementia must protect privacy and acknowledge their vulnerability to data sharing and fraud.	97.87%
Technology should support the autonomy of people living with dementia, allowing them to make decisions on what is comfortable for them while ensuring safety.	97.87%
Care professionals and researchers should ensure equal access to technology, take socioeconomic and cultural factors into account, and improve accessibility and sustainability.	95.74%
Technology in dementia care must meet best practices for ethical standards and regulations for fair use as well as artificial intelligence. Researchers and developers must ensure this happens during design and development.	95.74%

## 4 DISCUSSION

This study used a Delphi method to reach a consensus on 23 key statements regarding the development, usability, cost‐effectiveness, implementation, and ethics of ATs for people living with dementia and caregivers. Our results highlight the need for a user‐centered approach in the development of AT for people living with dementia and caregivers. A strong consensus emerged on the importance of involving people living with dementia and caregivers in research and decision‐making to ensure ATs align with real‐life needs. Integrating ATs into care plans at appropriate stages, whether at home or in a care facility, was also identified as a critical factor in usability and acceptance. The formulated statements reflect a consensus on key areas for the advancement of dementia‐related technologies and support.

There has been a significant rise in ATs for dementia, with innovation accelerating notably in 2019.^21^ ATs are increasingly used to enhance the well‐being of people living with dementia although dementia care guidelines across Europe currently fail to recommend specific ATs as part of holistic care.^22^ Our study reaffirms many of the recommendations from the 2017 position paper,^15^ indicating that key challenges remain unresolved. By updating these challenges into recommendations that reflect current perspectives and needs—while incorporating insights from people living with dementia, caregivers, healthcare professionals, and technology developers—we have refined previous findings to ensure their relevance in today's technological landscape. Moving forward, these updated priorities should guide future research, policy, and practice, ensuring that technology effectively supports people living with dementia and caregivers.

Core principles in the development of ATs include collaboration and accessibility. Collaboration between technology developers and end‐users was widely supported by the participants of this Delphi study. The participants stated the importance of understanding diverse user needs and experiences to design inclusive ATs solutions that improve daily life for people living with dementia and caregivers. They emphasized the need for developing adaptable, simple, and practical solutions to drive timely and effective delivery of AT to support people living with dementia. Chien et al.^23^ highlighted in their review that usability is essential in technological interventions for people living with dementia and caregivers, requiring attention to user characteristics, disease progression, sensory impairments, and age‐related changes. Technology should evolve alongside symptoms, offering versatile, multifunctional designs and personalized software functionalities. Challenges identified in open‐text responses to our Delphi survey underscored the necessity of balancing technological advancements with ease of use. Assistive technologies should not overwhelm people living with dementia and caregivers with complexity. To promote equal access and adoption, AT developers and providers should support open‐source development, allowing AT tools to be copied and shared among developers and users. Our findings also highlighted that defining costs and benefits from the outset is essential so that the AT tools are worthwhile. Previously, Boyle et al.^8^ recommended to address barriers such as the digital divide and disparities in digital health literacy, both of which significantly impact adoption. A recent review by Wolff et al.^24^ found that most research on dementia and technology has been conducted in high‐income countries. To promote equitable access and improve care for people living with dementia, the authors advocate for the use of equity frameworks—such as the Digital Health Equity Framework^25^ in the development of AT. They further emphasize the importance of expanding research efforts in low‐ and middle‐income countries to better align with global equity goals.^24^ This recommendation also extends to minority groups, who remain significantly underrepresented in technology‐related dementia research.

Based on the results of this Delphi study, future efforts could follow a structured roadmap to ensure accessibility of ATs for people living with dementia and caregivers to support daily living. This roadmap should aim to support daily living while ensuring inclusivity and usability. Several existing frameworks provide valuable guidance. For instance, the DEDICATE architecture^26^ emphasizes coordinated collaboration between formal and informal caregivers, supported by an affordable technological infrastructure. Another notable framework is REAFF^27^, which outlines four guiding principles for developing dementia‐friendly technologies: responding, enabling, augmenting and failure‐free. While these frameworks provide robust foundations, the domains of development, usability, cost‐effectiveness, implementation, and ethics, identified by Meiland et al.^15^ for application areas of ATs remain highly relevant and should continue to guide development efforts. In the short term, the development and usability of ATs should prioritize involving people living with dementia and caregivers from the outset, utilizing participatory methods. This ensures that the technologies meet user needs and are practical for integration into daily care practices, supported by clear training and information for caregivers. Our participants highlighted the need for fostering collaboration and training stakeholders to improve their understanding of the lived‐experiences of people living with dementia and caregivers and how these experiences drive their needs. Developing a common vision for ATs, such as how ATs might look like in the future and what they can offer (including the potential of AI), could help setting realistic expectations for future AT development and implementation. Another key benefit of collaboration between end‐users and technology developers is to ensure adaptability, while also addressing affordability and accessibility in high‐risk or underserved care environments. Ethical principles, particularly regarding autonomy and consent, must be integrated into AT development from the outset. It is essential to work towards high quality and cost‐effective technology to deliver ethical standards throughout the development and use of ATs to support people living with dementia and caregivers. Future priorities should focus on expanding research to include low‐ and middle‐income countries and underrepresented minority groups, promoting equity in dementia care. A structured roadmap for the deployment and scaling of ATs solutions is necessary, with a focus on overcoming digital illiteracy and bridging the digital divide to ensure broad and sustainable access. Additionally, further stakeholder engagement in follow‐up Delphi rounds or expert workshops can refine these priorities and create actionable implementation strategies. This approach should be guided by equity frameworks such as the Digital Health Equity Framework^25^ to ensure that ATs are both effective and inclusive in the long run.

### 4.1 Strengths and limitations

A strength of this study was the extensive involvement of PPI contributors as part of the independent research team throughout the research process. Their views and feedback on the conceptualization of the study were gathered from the outset, ensuring that the study reflected a broad range of perspectives and lived experiences. The study also adhered to the CREDES guidelines for conducting and reporting Delphi studies, enhancing its methodological rigor. The diverse group of respondents, representing multiple European countries and various fields, including people living with dementia and caregivers, healthcare professionals, researchers, and technology developers, offered a comprehensive and realistic perspective on dementia‐related technology on a European level. However, this study is not without limitations. A high dropout rate in round 2 may have affected the comprehensiveness and representativeness of later responses. Retention rates in Delphi studies are inconsistent. Previous international Delphi surveys, involving multi‐stakeholder groups including healthcare users, report retention rates between 19.5% and 87.1%.^28^ Additionally, despite the extensive efforts of the research team and supporting stakeholders in circulating the survey links in relevant networks to increase the diversity of participants, the involvement of people with dementia and technology developers in this study was lower than desired. This limitation emphasizes the value of and need to develop stronger collaboration between researchers, care providers, end‐users, and the tech industry so that their experiences are considered when developing and implementing technology. Such interdisciplinary partnerships are crucial to ensuring that technological solutions are grounded in the lived experiences and needs of people living with dementia and caregivers. Another limitation is about language complexity in dementia research that involves people with lived experiences. Despite strong PPI involvement and efforts to use a plain survey language, some Delphi statements were challenging for people living with dementia and caregivers to understand as mentioned in the open‐text comments in round 1. This experience highlights the essential importance and necessity of greater linguistic accessibility in dementia research tools. Although the research team particularly worked on using simplified English language in the surveys to promote diverse participation, the majority of the participants were from English‐speaking countries. This limits the diversity in participation and representativeness of our findings, underscoring the importance of broader international engagement in future research. Achieving this requires sufficient funding to allow for translation of the Delphi study into various European languages and extensive recruitment across diverse countries. The final limitation is the lack of real‐world technology evaluation. This study's scope was only limited to gathering and presenting expert opinions on AT development and use to support people living with dementia and caregivers rather than testing specific technologies in real‐world settings, which may limit the immediate applicability of the study results. Future studies are needed to test the application of co‐designed ATs based on the recommendations developed in this study. Nevertheless, this study offers valuable insights at the European level, highlighting the need for cross‐country research funding to address common gaps. It also provides recommendations for future dementia technology development that could be coordinated on a European scale.

## 5 CONCLUSION

This Delphi study emphasizes the importance of co‐designing ATs with people living with dementia and caregivers to ensure relevance and usability. Addressing accessibility, affordability, and the digital divide should be key priorities in the development of dementia‐supportive technology. Future research should strive for greater inclusivity, particularly by involving technology developers and people living with dementia and caregivers. Participation should be expanded across diverse linguistic and cultural contexts. Aligning research priorities with lived experiences will drive meaningful improvements in dementia care and support.

## CONFLICT OF INTEREST STATEMENT

The authors declare no conflicts of interest. Author disclosures are available in the Supporting Information.

## CONSENT STATEMENT

All human subjects provided informed consent.

## Supporting information



Supporting information

Supporting information

Supporting information
