# Supplementary material for: Recommendations for the development and use of technology to support people living with dementia and caregivers: A Delphi study
Source: Alzheimers Dement. 2025 Sep 29;21(10):e70755. doi: 10.1002/alz.70755 (PMC12477490; doi:10.1002/alz.70755)
Supplement: Supplementary file 2 — Supporting information [file ALZ-21-e70755-s003.docx]

**Appendix A**

**Round 1 statement rankings**

| **Development of technology** | | | | | | | | | | | | | | | | | | | | | | | | | | |  |  |  |  |
| --- | --- | --- | --- | --- | --- | --- | --- | --- | --- | --- | --- | --- | --- | --- | --- | --- | --- | --- | --- | --- | --- | --- | --- | --- | --- | --- | --- | --- | --- | --- |
| **Statement** | **Agreement rate** | | **(5) Strongly agree** | | **(4) Agree** | **(3) Neutral** | | | **(2) Disagree** | | | | **(1) Strongly disagree** | | | | **I do not have an opinion** | | | | | **Overall** | | | | |  |  |  |  |
| People living with dementia should be central to creating technology to support them in their lives. | 88.68% agree or strongly agree | | 65 | | 29 | 7 | | | 4 | | | | 1 | | | | 0 | | | | | 106 | | | | |  |  |  |  |
|  |  |  | 61.32% | | 27.36% | 6.60% | | | 3.77% | | | | 0.94% | | | | 0.00% | | | | | 100.00% | | | | |  |  |  |  |
| Tools should be personal and tailored to the needs of people living with dementia. | 94.34% agree or strongly agree | | 68 | | 32 | 4 | | | 2 | | | | 0 | | | | 0 | | | | | 106 | | | | |  |  |  |  |
|  |  |  | 64.15% | | 30.19% | 3.77% | | | 1.89% | | | | 0.00% | | | | 0.00% | | | | | 100.00% | | | | |  |  |  |  |
| Tools need to be in the homes of people with dementia and part of their care routines. | 78.30% agree or strongly agree | | 50 | | 33 | 17 | | | 3 | | | | 2 | | | | 1 | | | | | 106 | | | | |  |  |  |  |
|  |  |  | 47.17% | | 31.13% | 16.04% | | | 2.83% | | | | 1.89% | | | | 0.94% | | | | | 100.00% | | | | |  |  |  |  |
| Researchers and technology developers should get special training to understand the needs of people with dementia. | 96.23% agree or strongly agree | | 77 | | 25 | 0 | | | 3 | | | | 1 | | | | 0 | | | | | 106 | | | | |  |  |  |  |
|  |  |  | 72.64% | | 23.58% | 0.00% | | | 2.83% | | | | 0.94% | | | | 0.00% | | | | | 100.00% | | | | |  |  |  |  |
| **User-friendliness of the technology** | | | | | | | | | | | | | | | | | | | | | | | | | | | |  |  |  |
| **Statement** | **Agreement rate** | **(5) Strongly agree** | | **(4) Agree** | | | | **(3) Neutral** | | | **(2) Disagree** | | | | **(1) Strongly disagree** | | | **I do not have an opinion** | | | | | **Overall** | | | | |  |  |  |
| Research should find out the needs of people living with dementia and how to address them. | 97.17% agree or strongly agree | 84 | | 19 | | | | 1 | | | 1 | | | | 1 | | | 0 | | | | | 106 | | | | |  |  |  |
|  |  | 79.25% | | 17.92% | | | | 0.94% | | | 0.94% | | | | 0.94% | | | 0.00% | | | | | 100.00% | | | | |  |  |  |
| Research is needed to find out how technology can help them live independently. | 92.45% agree or strongly agree | 62 | | 36 | | | | 7 | | | 1 | | | | 0 | | | 0 | | | | | 106 | | | | |  |  |  |
|  |  | 58.49% | | 33.96% | | | | 6.60% | | | 0.94% | | | | 0.00% | | | 0.00% | | | | | 100.00% | | | | |  |  |  |
| Research is needed so people living with dementia can use technology for independent living, no matter their background or education. | 87.74% agree or strongly agree | 71 | | 22 | | | | 5 | | | 5 | | | | 3 | | | 0 | | | | | 106 | | | | |  |  |  |
|  |  | 66.98% | | 20.75% | | | | 4.72% | | | 4.72% | | | | 2.83% | | | 0.00% | | | | | 100.00% | | | | |  |  |  |
| **Costs and benefits of the technology** | | | | | | | | | | | | | | | | | | | | | | | | | | | | |  |  |
| **Statement** | **Agreement rate** | **(5) Strongly agree** | | **(4) Agree** | | | **(3) Neutral** | | | **(2) Disagree** | | | | **(1) Strongly disagree** | | | | | **I do not have an opinion** | | | | | **Overall** | | | | |  |  |
| Researchers and developers should make technology affordable for people living with dementia. | 89.62% agree or strongly agree | 76 | | 19 | | | 8 | | | 0 | | | | 1 | | | | | 2 | | | | | 106 | | | | |  |  |
|  |  | 71.70% | | 17.92% | | | 7.55% | | | 0.00% | | | | 0.94% | | | | | 1.89% | | | | | 100.00% | | | | |  |  |
| Researchers should study if technology provides value for money. | 80.19% agree or strongly agree | 50 | | 35 | | | 11 | | | 4 | | | | 2 | | | | | 4 | | | | | 106 | | | | |  |  |
|  |  | 47.17% | | 33.02% | | | 10.38% | | | 3.77% | | | | 1.89% | | | | | 3.77% | | | | | 100.00% | | | | |  |  |
| Care professionals should look at how technology helps each person with dementia, because everyone is different and might have other health problems too. | 94.34% agree or strongly agree | 74 | | 26 | | | 4 | | | 0 | | | | 2 | | | | | 0 | | | | | 106 | | | | |  |  |
|  |  | 69.81% | | 24.53% | | | 3.77% | | | 0.00% | | | | 1.89% | | | | | 0.00% | | | | | 100.00% | | | | |  |  |
| Researchers and developers should find simple and quick ways to see if technology benefits people with dementia. | 84.91% agree or strongly agree | 60 | | 30 | | | 9 | | | 2 | | | | 3 | | | | | 2 | | | | | 106 | | | | |  |  |
|  |  | 56.60% | | 28.30% | | | 8.49% | | | 1.89% | | | | 2.83% | | | | | 1.89% | | | | | 100.00% | | | | |  |  |
| **Use of technology** | | | | | | | | | | | | | | | | | | | | | | | | | | | | | |  |
| **Statement** | **Agreement rate** | **(5) Strongly agree** | | **(4) Agree** | | | **(3) Neutral** | | | **(2) Disagree** | | | | | **(1) Strongly disagree** | | | | | **I do not have an opinion** | | | | | **Overall** | | | | |  |
| Lack of information and knowledge is a barrier for people living with dementia and caregivers to use technology. | 87.74% agree or strongly agree | 57 | | 36 | | | 6 | | | 1 | | | | | 2 | | | | | 4 | | | | | 106 | | | | |  |
|  |  | 53.77% | | 33.96% | | | 5.66% | | | 0.94% | | | | | 1.89% | | | | | 3.77% | | | | | 100.00% | | | | |  |
| Technology does not always fit well in the current healthcare practices and this is a barrier to its use. | 74.53% agree or strongly agree | 48 | | 31 | | | 14 | | | 10 | | | | | 1 | | | | | 2 | | | | | 106 | | | | |  |
|  |  | 45.28% | | 29.25% | | | 13.21% | | | 9.43% | | | | | 0.94% | | | | | 1.89% | | | | | 100.00% | | | | |  |
| Care professionals should support people with dementia and caregivers to learn how to use technology if they need it. | 83.02% agree or strongly agree | 50 | | 38 | | | 17 | | | 0 | | | | | 1 | | | | | 0 | | | | | 106 | | | | |  |
|  |  | 47.17% | | 35.85% | | | 16.04% | | | 0.00% | | | | | 0.94% | | | | | 0.00% | | | | | 100.00% | | | | |  |
| Researchers and developers should plan how technology will be set up and used, for example who will install it and who will teach people with dementia how to use it. | 86.79% agree or strongly agree | 70 | | 22 | | | 7 | | | 3 | | | | | 2 | | | | | 2 | | | | | 106 | | | | |  |
|  |  | 66.04% | | 20.75% | | | 6.60% | | | 2.83% | | | | | 1.89% | | | | | 1.89% | | | | | 100.00% | | | | |  |
| **Ethical or fair use of technology** | | | | | | | | | | | | | | | | | | | | | | | | | | | | | | |
| **Statement** | **Agreement rate** | **(5) Strongly agree** | | **(4) Agree** | | | **(3) Neutral** | | | | | **(2) Disagree** | | | | **(1) Strongly disagree** | | | | | **I do not have an opinion** | | | | | **Overall** | | | | |
| Technologies need to be developed to protect and maintain the privacy of the person with dementia. | 89.62% agree or strongly agree | 64 | | 31 | | | 8 | | | | | 0 | | | | 2 | | | | | 1 | | | | | 106 | | | | |
|  |  | 60.38% | | 29.25% | | | 7.55% | | | | | 0.00% | | | | 1.89% | | | | | 0.94% | | | | | 100.00% | | | | |
| Technologies should be designed to help people with dementia make their own choices while also keeping them safe. | 91.51% agree or strongly agree | 68 | | 29 | | | 5 | | | | | 2 | | | | 2 | | | | | 0 | | | | | 106 | | | | |
|  |  | 64.15% | | 27.36% | | | 4.72% | | | | | 1.89% | | | | 1.89% | | | | | 0.00% | | | | | 100.00% | | | | |
| Care professionals and researchers shouldn't just talk about the fair use of technology; they should apply this to their practice. | 83.96% agree or strongly agree | 58 | | 31 | | | 9 | | | | | 1 | | | | 2 | | | | | 5 | | | | | 106 | | | | |
|  |  | 54.72% | | 29.25% | | | 8.49% | | | | | 0.94% | | | | 1.89% | | | | | 4.72% | | | | | 100.00% | | | | |
| Researchers and developers should follow standards and guidelines about the ethical and fair use of technology to support people with dementia. | 92.45% agree or strongly agree | 77 | | 21 | | | 5 | | | | | 1 | | | | 1 | | | | | 1 | | | | | 106 | | | | |
|  |  | 72.64% | | 19.81% | | | 4.72% | | | | | 0.94% | | | | 0.94% | | | | | 0.94% | | | | | 100.00% | | | | |

**Round 2 statement rankings**

| **Development of technology** | | | | | | | | | | | | | | | | | | | | | |  |  |
| --- | --- | --- | --- | --- | --- | --- | --- | --- | --- | --- | --- | --- | --- | --- | --- | --- | --- | --- | --- | --- | --- | --- | --- |
| Statement | **Agreement rate in Round 1** | **Agreement rate in Round 2** | **(5) Strongly agree** | | **(4) Agree** | | | **(3) Neutral** | | | **(2) Disagree** | | | **(1) Strongly disagree** | | | **I do not have an opinion** | | **Overall** | | |  |  |
| **(Amended based on the comments provided in Round 1)** People living with dementia and caregivers must be at the forefront at all stages of the design and development of technology to support them. | 88.68% agree or strongly agree | 95.74% agree or strongly agree | 30 | | 15 | | | 2 | | | 0 | | | 0 | | | 0 | | 47 | | |  |  |
|  |  |  | 63.83% | | 31.91% | | | 4.26% | | | 0.00% | | | 0.00% | | | 0.00% | | 100.00% | | |  |  |
| **(Amended based on the comments provided in Round 1)** Tools should be customisable to each specific stage of dementia to address the changing needs of people living with dementia and caregivers. | 94.34% agree or strongly agree | 91.49% agree or strongly agree | 29 | | 14 | | | 3 | | | 1 | | | 0 | | | 0 | | 47 | | |  |  |
|  |  |  | 61.70% | | 29.79% | | | 6.38% | | | 2.13% | | | 0.00% | | | 0.00% | | 100.00% | | |  |  |
| **(Amended based on the comments provided in Round 1)** Tools should be provided to people living with dementia as part of their care plan and incorporated in their daily care routines and at the appropriate time to their needs. | 78.30% agree or strongly agree | 85.11% agree or strongly agree | 29 | | 11 | | | 7 | | | 0 | | | 0 | | | 0 | | 47 | | |  |  |
|  |  |  | 61.70% | | 23.40% | | | 14.89% | | | 0.00% | | | 0.00% | | | 0.00% | | 100.00% | | |  |  |
| **(Amended based on the comments provided in Round 1)** Researchers and technology developers must learn about the needs of people living with dementia by including them in the research and design process. | 96.23% agree or strongly agree | 97.87% agree or strongly agree | 40 | | 6 | | | 1 | | | 0 | | | 0 | | | 0 | | 47 | | |  |  |
|  |  |  | 85.11% | | 12.77% | | | 2.13% | | | 0.00% | | | 0.00% | | | 0.00% | | 100.00% | | |  |  |
| **(New statement)** Tools should be designed to complement human interaction, not replace it. | NA | 95.74% agree or strongly agree | 39 | | 6 | | | 2 | | | 0 | | | 0 | | | 0 | | 47 | | |  |  |
|  |  |  | 82.98% | | 12.77% | | | 4.26% | | | 0.00% | | | 0.00% | | | 0.00% | | 100.00% | | |  |  |
| **(New statement)** Collaboration across health and social care practitioners and researchers, technology developers, people living with dementia and caregivers is essential to create effective and accessible technologies | NA | 100% agree or strongly agree | 38 | | 9 | | | 0 | | | 0 | | | 0 | | | 0 | | 47 | | |  |  |
|  |  |  | 80.85% | | 19.15% | | | 0.00% | | | 0.00% | | | 0.00% | | | 0.00% | | 100.00% | | |  |  |
| **User-friendliness of the technology** | | | | | | | | | | | | | | | | | | | | | | | |
| **Statement** | **Agreement rate in Round 1** | **Agreement rate in Round 2** | | **(5) Strongly agree** | | **(4) Agree** | | | **(3) Neutral** | | | **(2) Disagree** | | | **(1) Strongly disagree** | | | **I do not have an opinion** | | | **Overall** | | |
| **(No further contributions were found in the comments. Agreed, no changes)** Research should find out the needs of people living with dementia and how to address them. | 97.17% agree or strongly agree | NA | | NA | | | | | | | | | | | | | | | | | | | |
| **(No further contributions were found in the comments. Agreed, no changes)** Research is needed to find out how technology can help them live independently. | 92.45% agree or strongly agree | NA | | NA | | | | | | | | | | | | | | | | | | | |
| **(No further contributions were found in the comments. Agreed, no changes)** Research is needed so people living with dementia can use technology for independent living, no matter their background or education. | 87.74% agree or strongly agree | NA | | NA | | | | | | | | | | | | | | | | | | | |
| **(New statement)** Researchers and developers must recognise that many people living with dementia and their caregivers have limited experience with technology. | NA | 85.11% agree or strongly agree | | 24 | | 16 | | | 4 | | | 3 | | | 0 | | | 0 | | | 47 | | |
|  |  |  |  | 51.06% | | 34.04% | | | 8.51% | | | 6.38% | | | 0.00% | | | 0.00% | | | 100.00% | | |
| **(New statement)** It is essential to design and develop technology that is simple and accessible to meet their needs and enhance their quality of life. | NA | 97.87% agree or strongly agree | | 39 | | 7 | | | 1 | | | 0 | | | 0 | | | 0 | | | 47 | | |
|  |  |  |  | 82.98% | | 14.89% | | | 2.13% | | | 0.00% | | | 0.00% | | | 0.00% | | | 100.00% | | |
| **Costs and benefits of the technology** | | | | | | | | | | | | | | | | | | | | | | |  |
| **Statement** | **Agreement rate in Round 1** | **Agreement rate in Round 2** | | **(5) Strongly agree** | | **(4) Agree** | | | **(3) Neutral** | | | **(2) Disagree** | | | **(1) Strongly disagree** | | | **I do not have an opinion** | | **Overall** | | |  |
| **(No further contributions were found in the comments. Agreed, no changes)** Researchers and developers should make technology affordable for people living with dementia. | 89.62% agree or strongly agree | NA | | NA | | | | | | | | | | | | | | | | | | |  |
| **(No further contributions were found in the comments. Agreed, no changes)** Researchers should study if technology provides value for money. | 80.19% agree or strongly agree | NA | | NA | | | | | | | | | | | | | | | | | | |  |
| **(Amended based on the comments provided in Round 1)** Dementia manifests itself differently for each person but everyone goes through similar stages. Technology needs to support the specific needs of people living with dementia at each stage of the disease. | 94.34% agree or strongly agree | 91.49% agree or strongly agree | | 22 | | 21 | | | 3 | | | 0 | | | 1 | | | 0 | | 47 | | |  |
|  |  |  |  | 46.81% | | 44.68% | | | 6.38% | | | 0.00% | | | 2.13% | | | 0.00% | | 100.00% | | |  |
| **(Amended based on the comments provided in Round 1)** Researchers and developers should develop clear criteria to validate the effectiveness of technology to benefit people living with dementia and caregivers (such as helping them stay connected or manage day-to-day tasks). | 84.91% agree or strongly agree | 91.49% agree or strongly agree | | 30 | | 13 | | | 4 | | | 0 | | | 0 | | | 0 | | 47 | | |  |
|  |  |  |  | 63.83% | | 27.66% | | | 8.51% | | | 0.00% | | | 0.00% | | | 0.00% | | 100.00% | | |  |
| **(New statement)** Technology should ease caregiver responsibilities, not increase them. | NA | 95.74% agree or strongly agree | | 35 | | 10 | | | 2 | | | 0 | | | 0 | | | 0 | | 47 | | |  |
|  |  |  |  | 74.47% | | 21.28% | | | 4.26% | | | 0.00% | | | 0.00% | | | 0.00% | | 100.00% | | |  |
| **Use of technology** | | | | | | | | | | | | | | | | | | | | | |  |  |
| **Statement** | **Agreement rate in Round 1** | **Agreement rate in Round 2** | | **(5) Strongly agree** | | **(4) Agree** | | | **(3) Neutral** | | | **(2) Disagree** | | | **(1) Strongly disagree** | | | **I do not have an opinion** | **Overall** | | |  |  |
| **(No further contributions were found in the comments. Excluded)** Technology does not always fit well in the current healthcare practices and this is a barrier to its use. | 74.53% agree or strongly agree | NA | | NA | | | | | | | | | | | | | | | | | |  |  |
| **(Amended based on the comments provided in Round 1)** It is essential to provide education for people living with dementia and caregivers and train care staff about how to use the technology. A lack of information and knowledge is a barrier to the use of technology. | 87.74% agree or strongly agree | 95.74% agree or strongly agree | | 32 | | 13 | | | 2 | | | 0 | | | 0 | | | 0 | 47 | | |  |  |
|  |  |  |  | 68.09% | | 27.66% | | | 4.26% | | | 0.00% | | | 0.00% | | | 0.00% | 100.00% | | |  |  |
| **(Amended based on the comments provided in Round 1)** It is important that experts are available to provide individual, personalised support during installation and continued use of the technology (such as dedicated end-user support and providing training through community services). | 83.02% agree or strongly agree | 95.74% agree or strongly agree | | 30 | | 15 | | | 2 | | | 0 | | | 0 | | | 0 | 47 | | |  |  |
|  |  |  |  | 63.83% | | 31.91% | | | 4.26% | | | 0.00% | | | 0.00% | | | 0.00% | 100.00% | | |  |  |
| **(Amended based on the comments provided in Round 1)** Researchers and developers should focus on who will use technology in everyday environments to ensure it meets user needs, integrates into routines and is compatible. | 86.79% agree or strongly agree | 97.87% agree or strongly agree | | 37 | | 9 | | | 1 | | | 0 | | | 0 | | | 0 | 47 | | |  |  |
|  |  |  |  | 78.72% | | 19.15% | | | 2.13% | | | 0.00% | | | 0.00% | | | 0.00% | 100.00% | | |  |  |
| **Ethical or fair use of technology** | | | | | | | | | | | | | | | | | | | | | |  |  |
| **Statement** | **Agreement rate in Round 1** | **Agreement rate in Round 2** | | **(5) Strongly agree** | | **(4) Agree** | **(3) Neutral** | | | **(2) Disagree** | | | **(1) Strongly disagree** | | | **I do not have an opinion** | | | **Overall** | | |  |  |
| **(Amended based on the comments provided in Round 1)** Technologies for people living with dementia must protect privacy and acknowledge their vulnerability to data sharing and fraud. | 89.62% agree or strongly agree | 97.87% agree or strongly agree | | 41 | | 5 | 1 | | | 0 | | | 0 | | | 0 | | | 47 | | |  |  |
|  |  |  |  | 87.23% | | 10.64% | 2.13% | | | 0.00% | | | 0.00% | | | 0.00% | | | 100.00% | | |  |  |
| **(Amended based on the comments provided in Round 1)** Technology should support the autonomy of people living with dementia, allowing them to make decisions on what is comfortable for them while ensuring safety. | 91.51% agree or strongly agree | 97.87% agree or strongly agree | | 37 | | 9 | 1 | | | 0 | | | 0 | | | 0 | | | 47 | | |  |  |
|  |  |  |  | 78.72% | | 19.15% | 2.13% | | | 0.00% | | | 0.00% | | | 0.00% | | | 100.00% | | |  |  |
| **(Amended based on the comments provided in Round 1)** Care professionals and researchers should ensure equal access to technology, take socioeconomic and cultural factors into account, and improve accessibility and sustainability. | 83.96% agree or strongly agree | 95.74% agree or strongly agree | | 29 | | 16 | 2 | | | 0 | | | 0 | | | 0 | | | 47 | | |  |  |
|  |  |  |  | 61.70% | | 34.04% | 4.26% | | | 0.00% | | | 0.00% | | | 0.00% | | | 100.00% | | |  |  |
| **(Amended based on the comments provided in Round 1)** Technology in dementia care must meet best practices for ethical standards and regulations for fair use as well as artificial intelligence. Researchers and developers must ensure this happens during design and development. | 92.45% agree or strongly agree | 95.74% agree or strongly agree | | 38 | | 7 | 2 | | | 0 | | | 0 | | | 0 | | | 47 | | |  |  |
|  |  |  |  | 80.85% | | 14.89% | 4.26% | | | 0.00% | | | 0.00% | | | 0.00% | | | 100.00% | | |  |  |
