# Supplementary material for: Recommendations for the development and use of technology to support people living with dementia and caregivers: A Delphi study
Source: Alzheimers Dement. 2025 Sep 29;21(10):e70755. doi: 10.1002/alz.70755 (PMC12477490; doi:10.1002/alz.70755)
Supplement: Supplementary file 3 — Supporting information [file ALZ-21-e70755-s001.docx]

| **Round 1 statement** | **Agreement rate (n=106 participants)** | **Relavant open-text comments** | **Suggested edits to this statement to address the feedback** |
| --- | --- | --- | --- |
| **Development of technology** | | | |
| People living with dementia should be central to creating technology to support them in their lives. | 88.68% agree or strongly agree | - Consult people with lived experience about our needs (26073098) - involve pLWD and Care partners and family members at all stages (25977236) - […] Also PPI involvement in projects is not always set up properly, resulting in innovations that are not well accepted in daily practice. (25937856) - Develop user-centred design practice […] (25327554) - […] People with dementia and their caregivers should participate in the pilots and trials of new technology developments. (25843659) - Ask people as you go along with each step of the process. (26055967) - ask people what they want […] Tailor individual needs after proper assessment (26055869) - involve people living with dementia in determining the path forward (25942520) - Work with PLWD and their partners in care in the developing stage just ask us (26114813) - The voice of the lived experience should always be front and central to everything about them (25283800) - Use people with dementia to design and disseminate information, we trust folk with Livd Experience more than Academics (26057431) - people living with dementia should be involved in every stage of any technology research aim to give us what we want and need never presume what we want or give us something you think will be useful to us in our lives please please ask and consult with us thankyou (26055869) - Get the input of people with dementia, their families, caregivers and healthcare professional as early in the process as you can and make the process as collaborative as you can throughout the research and development. (26055432) - the development of technology for people with dementia rarely includes them at the early development stage, which is too late (25977236) - listen to the people involved in the care process (25369379) - The views of people with dementia should be encouraged as much as possible and researchers should not rely on the testimony of carers. We should be exploring what people with dementia actually want from technology, what is desirable, and why - not a carer view. […] (25942471) - Good engagement between researchers and dementia groups… like this!! (26099123) - The development teams should include the view of all stakeholders such as PwD, carers etc. (24951005) - co-create with people living with dementia and their caregivers, make sure the technologie is what they need and is in line with how they would use it. (25572212) - Dont patronise, eg with dogs and cats and toys that interact, except for those late in their disease who can benefit. Have plwds involved from the start, ie before you evolve something to test. Observe/record daily living of plwds to find out through research what could help in real life. Dont make assumptions.(25448376) - Involve stakeholders (especially people with dementia and their carers) insights during the development process. […] (25571274) - Priorities for the purpose of the technology should be decided by people with dementia - this could be to support and maintain independence but may be for other purposes: meaningful activity; stimulation; connection (25942679) - Invest time and funds to work alongside plwds to develop tools and approaches. We will tell you if something will not work for us before you make it! (25448376) - Develop user-centred design practice where people living with dementia are involved in the identification of problems as well as the solutions. (25327554) - Ensure that people being used to test technology have a valid diagnosis and have dementia (25475638) - The fact that the dementia patient will not go with it or to that degree the carer. To ensure that there is an advancement but within it simplification, but if early onset dementia then everything should be tried to help them. Never presume anything until you have seen the individual so in essence visit the person/carer to then adapt your technology. (26095533) - During development, researchers must listen to: patients with dementia, caregivers and health and social professionals with experience in the area. Do not forget patients and caregivers from the lowest social gradients. Because they are the ones with the most unmet needs and the worst digital literacy. Finally, test the usability of the instruments. (25001442) - involve researchers, practitioners and people with dementia from third world countries (25942679) - The will to prioritise and address this issue The piecemeal approach to tresearch. Understanding the needs of people with dementia Lack of collaboration, commitment and leadership Finance (25212676) | **New aspects**   - Technologies should be developed in collaboration with people living with dementia and their caregivers to ensure that they meet actual needs and are tailored to their application. - Involve people living with dementia and caregivers at all stages of development starting from the beginning to creating technology to actually support them in their lives. - Don´t make assumptions about what people living with dementia need, the purpose of technology should be decided by the people living with dementia - Involve people with different backgrounds (socio-economic, diversity)   **Agreed amendment:**  People living with dementia and caregivers must be at the forefront at all stages of design and development of technology to support them. |
| Tools should be personal and tailored to the needs of people living with dementia. | 94.34% agree or strongly agree | - Better understand the needs and design with tailored needs. […] (25571274) - I found some if the technology that was available was totally unsuitable for Deminta patients […] (26060421) - Technology should be adaptable and be able to be changed by the caregiver (or someone) to fit the needs and interests of the person with dementia (25363444) - […] The partnership should be bespoke to accomodate each client’s needs, respecting their fears and wishes. (26057462) - Leave it open to adaptations (25357467) - Realise that dementia occurs in different ways for different people … (25317809) - Tailor-made hardware (25023763) - Incorporate AI to learn the unique needs of individuals (25279026) - Technologies should be flexible, learn from user behavior, and adjust their interface according to the individual's progression. […] (25861755) - […] Also not only important to consider individual needs of people living with dementia, but also that their needs change over time due to changes in abilities, interests, environment, etc. (24957622) - Dementia manifests in different ways and at different stages … (25317809) - To be abble to answer to individual needs of caregivers and patients, without being intrusive and expensive (25590900) - personalized design (25364119) - To tailor them to individual and family needs, and not to consider them a one-size fits all solution. (25079889) - Dementia affects individuals in different ways (cognitive decline, physical abilities, and emotional states). Designing technology that can adapt to these changing needs over time is difficult. […] (25861755) - The progressive nature of the disease and the need for a quick update on their function should be considered when designing them. (26056147) - […] It being appropriate to the individual as dementia presents itself in many different ways and can progress faster than the assistive technology can adapt. The ability to use new technology has be lost in the early stages of dementia before obvious symptoms. (26055432)   **Caregivers:**   - Technology should not only be used for the person with dementia (as key user) but also for the (in)formal caregivers (which may help to assist caring or supprot their daily lives) (24951560) - The caregivers have to be able to work with the technology, and they may not have time and willingness to add this to their long list of caring duties. (25330345) - Technology for dementia is not always used by the people with dementia but sometimes by caregivers (eg in managing the care, etc), family members, etc. This survey perhaps should differentiate between different use cases. (25317809) - Also the needs of family carers are important and they not always align with those of the person with dementia. One might want to provide recommendations in these cases. (26109408) - Ensure a comprehensive assessment is made of the person's needs, preference and abilities. Engage with their support network as to how best to use it and assist in making it effective. (26055432) | **New aspects**   - Needs change over time - needs vary from person to person - technologies should be customisable/adaptable to these diverse needs in order to be relevant and useful. - Adaptation to user behaviour (e.g. by using AI) - Flexible, adaptive technology is difficult to design. - Technology should include the needs of caregivers as they often assist or use the technology.   **Agreed amendment:**  Tools should be customisable to each specific stage of dementia to address the changing needs of people living with dementia and caregivers. |
| Tools need to be in the homes of people with dementia and part of their care routines. | 78.30% agree or strongly agree | - […] Development for technology fo help people with dementia should focus primarily one the person at home. Development of technology should not prioritise replacing staff in care homes. […] (25212676) - build technology use into ACP conversations (26090971) - […] embed them into care pathways. […] (25517807) - Engage at every level. Eg when first “diagnosis” is being given, there should ALWAYS be the inclusion of technology as an aspect of care (even if its not immediately available) (26071806) - […] Try to normalise the use of technology so that it is not seen as something required specifically or the condition but something that is beneficial for overall wellbeing (e.g. the way the use of fitbits, smart watches etc, has become normalised). Need to consider who the tech is really for - the person with dementia or the (in)formal carer and proceed accordingly […] (25517807) | **Team discussions and PPI input:**  Tools should be embedded in the care pathway and in discussions on advanced care planning from the very beginning.  **Agreed amendment:**  Tools should be provided to people living with dementia as part of their care plan and incorporated in their daily care routines and at the appropriate time to their needs. |
| Researchers and technology developers should get special training to understand the needs of people with dementia. | 96.23% agree or strongly agree | - […] About being trained in the needs of persons with dementia: this depends on the competencies of researchers and technology developers. […] (26109408) | **Team discussions and PPI input:**  With support of PPI and the benefit of lived experience.  **Agreed amendment:**  Researchers and technology developers must learn about the needs of people living with dementia by including them in the research and design process. |
| New statement  Tools should be designed to complement, not replace human interaction. | NA | - […]. • Technologies should be designed to complement, not replace, human interaction. For instance, tools that facilitate social engagement, connect PwD with family and friends, or support in-person care should be prioritized. […] (25861755) - understanding technology complimentary to human work, […] (25937062) - Current questions I have had raised through my work are concerns about the wider implications of technology such as AI and robotics and how these risk having negative impacts on society (eg. replacing humans, reducing work availability, safety, closing of banks/shops) - this can be a barrier to people wishing to engage in technology. (25942471) - […] the wider implications of technology […] having negative impacts on society (eg. replacing humans, […]. (25942471) - Every one should have the facilities to use technology no matter what their disability. We need to keep people connected. No one should feel alone and for some that are housebound technology is the only way to keep connected (26055892) - While technology can assist with monitoring, over-reliance on these tools could reduce human contact, leading to increased social isolation for individuals with dementia. […] (25861755) - Technology should not replace human day to day contact. It should start to be used early on in diagnosis to be most effective. Repeditive games etc can be very effective in stimulating the person who is in cognative decline (25500731) - That the due investment in new technological solutions does not overshadow the need to invest in human contact and support, including the family, friends and neighbours, and formal carers. (25079889) - I believe all technology has it’s limits, and should not be developed to exclude human care givers being involved. […] (26057462) - Research how technology can supplement existing services in a way that does not feel like technology is replacing services and people. […] (25942471) - […] increasing awareness (e.g. politicians) that increasing the use of everyday technology is costly (at least at first) AND still needs humans! (25937062) | **New aspects**  Tools should not replace human interaction.  **Agreed:**  Tools should be designed to complement human interaction, not replace it. |
| New statement  Interdisciplinary collaboration are necessary to create effective and accessible technologies. | NA | - Engage with startups and universities. (25460932) - More interdisciplinary collaboration between technology experts (human-computer interaction, human factors engineering and UX specialists) and experts in health care is needed; more holistic approaches are also needed, that consider digital ecosystems rather than just individual technologies in isolation. […] (24952169) - The link between carers, users and the developers of technology…. If researchers don’t understand the problem, they can design useful tech; […] (26099123) - Collaborate with industry partners to stay up to date, consider implementation from the start (25383836) - discuss with producers (25364119) - Close collaboration with industry, […] (25937062) - Good question! More collaboration between research and technology developers, flexibility in research design (24957622) - be receptive to everything. Maybe study tech in other countries eg Australia are the lead so what do they do. (26095533) - Firstly, conduct a rigorous literature review about existing technologies, identify the effective elements; explore people's experiences and perceptions of it, and potential challenges and solutions globally. Then, may need to do a qualitative interview with stakeholders to see if there are available resources to support the use of technologies. Meanwhile, invite patient and public involvement (PPI) contributors in the technology development process, to improve the relevance and effectiveness of the products. Moreover, it is necessary to contact policy makers/managers/directors of nursing homes, etc. to identify the potential conflicts of using technologies (25755060) - Make sure not to create something that already exists but rather work together and improve that already existing technology - more collaboration and less independent work of different universities/researchers. […] (25942808) | **New aspects**  Another challenge in designing useful technology is the lack of co-operation between developers of technology, research and those affected.  **Agreed:**  Collaboration across disciplines and communities is essential to create effective and accessible technologies (medical, industry, technical, social, people living with dementia and carers). |
| **User-friendliness of the technology** | | | |
| Research should find out the needs of people living with dementia and how to address them. | 97.17% agree or strongly agree | - […] Research should focus on understanding the needs of people living with dementia, understanding how technology works, identifying solutions to the needs of people living with dementia. (25212676) - I think the most important aspect is to explore the needs of people with dementia and their relatives/ informal caregivers. Is privacy an important topic for them, than technologies should also focus on that. But for some people it might not be important, and other technological interventions might be better. (25572212) - Think what you would find helpful if you have dementia. (26055967) - I am unqualified to make suggestions. I know so little about it. I expect that technologies will help some people greatly and be of little benefit for others. There is a need for greater research and developement. (25327042) | Agreed, no changes |
| Research is needed to find out how technology can help them live independently. | 92.45% agree or strongly agree | - […] Development of technology should focus on where it will be most effective, perhaps independent living.; […] (25212676) - If tech can talk to person and help give prompts to help themselves to give the dementia patient some feeling of independence without them having to rely on caregivers all the time (25326789) - Dementia is a cognative disability and suffers should have access to disability parking. Facial recognition technology screens should be developed for people living at home. An Electronic door tags something like those used in Aldi to display food prices should be developed for doors drawers and cubards inside the home to assist the person identify what is behind the closed door. (25331046) | Agreed, no changes |
| Research is needed so people living with dementia can use technology for independent living, no matter their background or education. | 87.74% agree or strongly agree | - i think one of the issues is how to incorporate certain groups of persons living with dementia: for instance those with difficulties accessing health care. They are the ones that should be included in the research, but that is often not the case (26031716) - […] find innovative ways to include people with low technology literacy and confidence into research. Most research is conducted on people with dementia or carers who already have some level of technology literacy, interest and confidence, more work needs to be done on finding those without technology skills for more valid representation. […] (25942471) | Agreed, no changes |
| New statement  Tools for people living with dementia and caregivers should be simple and user-friendly. / Tools should be as simple as possible in order to be user-friendly. |  | - To do technologies as simple as they can be (25590900) - […] Lack of simplicity. (25500731) - […] 2. Try to make the technologies more accessible, simple, and easy-to-use. (25571274) - Solutions need to be easy to use. (25460932) - Keep it simple. People with Dementia and their caregivers are already stressed & this makes it difficult to learn new skills. (25357467) - […] Dementia-friendly design and user-friendly design. […] (25094761) - Dementia=friendly design. Pilot use many times. (25094761) - Simplicity is the key (26063922) - […] make technology simple and very user friendly […] (26055869) - make it as easy to handle as possible […] (26031716) - Usability. And introducing thme to plwds at an early stage when they can learn to use them. […] (25448376) - […] Usability - must be straightforward - may also be a factor. (25327042) - Tech needs to be easy to use (26073098) - […] and not to be complicated. (26055967) - I think most challenging is to create technologies that fit the needs of different people, and technologies that are really easy to use both by people living with dementia and their caregivers. It is best when technologies meet the intuitive actions of people, or when less explaination is needed. (25572212) - Sensory deficits (25023763) - Capable of being used by an old person who on top of that is demented (25147560) | **Team discussions and PPI input:**  People living with dementia and their caregivers need simple and user-friendly tools.  **Agreed:**  Tools for people living with dementia and their caregivers should be designed to be as simple and user-friendly as possible. |
| New statement  Tools should help promote digital literacy. / Researchers and developers should consider the frequent lack of digital literacy. | NA | - Actions to promote mental health literacy and digital literacy (25983217) - […] Then, the e-health literacy may be another challenge, in general, people with dementia and caregivers are mostly older people, they may find it difficult to learn or use a new technology, especially with cognitive impairment. Above concerns may need to be considered in the future. (25755060) - New technologies including internet, apps and mobile phones can be a barrier to the use and adoption. (25460932) - digital illiteracy (25983217) - […] digital literacy for users and providers (25937062) | **New aspects**  Researchers and developers must be aware that people living with dementia and their carers, may not have knowledge or experience of using technology.  **Agreed:**  Researchers and developers must recognise the gap of knowledge or experience of using technology. |
| **Costs and benefits of the technology** | | | |
| Researchers and developers should make technology affordable for people living with dementia. | 89.62% agree or strongly agree | - It is as important to consider low-tech as well as high-tech solutions. It is also important to ensure that providers do not put a price premium on these technologies just because they see a new or expanding market. (25517807) - […] making them available at low cost or less. (25500731) - Make it carw centered not profit centerwd (26067329) - Ethical pricing, […] (25327042) - Try to keep costs low […] (25517807) - […] funding for technologies should also be considered from day one, (26056147) - […] The pan pan watch I felt was beneficial and gave peace of mind ..But really cost a lot of money which many could not afford (26060421) - Make these technologies affordable and increase literacy for its use. (24961604) - reasonable cost. […] (25843659) - Provide grants (25331046) - 1. Open source platforms of sensor technology 2. Reimbursement of technology (either consumer market or with financial support of municipalities or health insurances) […] (24951560) - Funding and accessibility (26055892)   **The costs of utilising technologies are perceived as a challenge.**   - Costs of technology, […] (25547050) - Cost/Price, […] (25517807) - Cost. Dementia radio 5 times the price of a regular radio. (25363444) - […] And cost. Probably cost effective to provide a newly diagnosed person living with dementia with an ipad and phone for example and train them to use them. (25448376) - Cost is always a challenge.[…] (25327042) - Development of technologies suitable for the sufferers that is grant supported by government. (25331046) - In addition to the cost, […] (25755060) - Money, understanding (26055892) - Affordability (26055916) - Finding low cost and suitability (26060421) - Cost, […] (26067329) - The size and cost, […] (26055967) - […] and cost (25977236) - The expense of equipment & trained people to demonstrate, teach & assist people using the technology. Also any further back up assistance. (26054921) - costs […] (26031716) - Financial challenges since it will commonly only be available to those who can afford it as insurance companies will probably not be interested in investing. […] (25942808) - […] costs of everyday technology, […] (25937062)   **Disagree**   - I don’t think it’s up to researchers and developers to necessarily ensure that tech is available, affordable, etc. they develop it, work it out, then others can roll it out, integrate it, etc. researchers shouldn’t be inhibited from best and innovative thinking by HSE budgets, etc […] | Agreed, no changes |
| Researchers should study if technology provides value for money. | 80.19% agree or strongly agree |  | Agreed, no changes |
| Care professionals should look at how technology helps each person with dementia, because everyone is different and might have other health problems too. | 94.34% agree or strongly agree | - Technology for people with dementia is very broad and each intervention needs to be focused: either on a specific stage of dementia or age group (25092956) - Technology needs to take the different stages of Dementia and the needs of the person at each stage into account […] (25212676) - For every person that has dementia whatever type that maybe, you must remember the ME in it. Every single person will have differences. Therefore common sense is needed. (26095533) - everyone is different… (25279026) - […] Exposure to real people with dementia is a big plus, because each person is different, and some of the symptoms are intriguing…. They would present challenges for researchers & developers, and lead to practical usable technology. (26099123) | **New aspects**  Not only care professionals, but also developers, researchers.  **Team discussions:**  Similar additions as for the 2. statement: “Tools should be personal and tailored to the needs of people living with dementia.”  **Agreed amendment:**  Dementia manifest itself differently for each person but everyone goes through the similar stages. Technology needs to support the specific needs of people living with dementia at each stage of the disease. |
| Researchers and developers should find simple and quick ways to see if technology benefits people with dementia. | 84.91% agree or strongly agree | - Please good faisability studies before introducing the technology (25369379) - It is of the utmost importance that the validation and demonstration of effectiveness are never overlooked or delayed when developing a device for the rehabilitation, care or supervision of a person with dementia. (25952113) - Learning from current practice is a good starting point - find out what the problems currently are (such as with telecare) and look for solutions. (25942520) - In general, I observe a lot of technology being implemented, eg in the nursing home, without *any* evidence for their effectiveness. Less focus on implementation, more on the scientific effects, please! (25447271) - Technologies need to have a perceived value and benefit (it's not always about autonomy or independence, it could also be communication, enjoyment, or physical activity). (25383836) - Based on my experiences, I can see some existing assistive technology for people with dementia and caregivers, but I'm wondering about the effectiveness and feasibility of those technology, is it necessary to develop a new one, or just optimise an existing one? How does people (people with dementia and caregivers, or practitioners) think about it, are they useful for them? I think this may be a priority as well before developing a technology? (25755060) - Recording & assessment systems also need to in place to keep record of technology efficacy & Aldo comments from the user. (26054921) - […] Research into the development of technology should be collaborative and include technologists from the outset. Research into technology to assist people living with dementia should be a public private partnership. Researchers should study on how the technology industry develops products and brings them to market. It will take an International commitment, funding and drive to bring assistive Technology to the people living with dementia. […] (25212676) - Maintaining an “open forum” which people could turn to with suggestions or specific requests for advice or practical help. This might then come more speedily and with more common sense than having to wait for the same advice to come from elsewhere (eg professionals) a good while later (26071806) - […] Finally, more effort is needed to understand the implementation of technology can impact it's use and benefits. Also, we need to explore how we are measuring the benefits (or not benefits) people with dementia are getting from these technologies. (25942471) - […] unknown benefits (26071806) | **New aspects**   - People living with dementia and caregiver must be involved - Instead of hasty implementations, the focus should be more on analysing the scientific effects of the technologies first.   **Agreed amendment:**  Researchers and developers should develop clear criteria to validate effectiveness of technology to benefit people living with dementia and caregivers (such as helping them staying connected or help manage day to day tasks).  . |
| New statement  Technology should reduce caregiver burden by prioritizing interoperability, intuitive design, and automated solutions that don't require constant supervision.  /  Technologies that are too complicated can lead to additional stress for people living with dementia and caregivers and increase the workload of caregivers. | NA | - […] • Technology should ease caregiver burden, not increase it. This requires a focus on interoperability, intuitive design, and solutions that automates tasks without the need of constant supervision. (25861755) - Look at each case individually. Decide whether the technology will help in the long term, or if it will become a hindrance and annoyance. Again, the beginning stages of dementia, the person / caregiver can handle the technology, but as the dementia progresses and worsens, these technologies will more likely be used by the caregivers and not the person suffering with the dementia. (25381891) - […] Do not add extra burden while using the technology or raise social isolation. (25571274) - […] Caregivers face heavy physical and emotional burdens. It is important to garantee that introducing new technologies will not inadvertently add to their workload (e.g., systems which are hard to manage or not integrated well with existing routines). Some technologies collect and display large amounts of data that can be overwhelming for caregivers and healthcare professionals to interpret. (25861755) | **Agreed:**  Technology should ease caregiver burden, not increase it. |
| **Use of technology** | | | |
| Lack of information and knowledge is a barrier for people living with dementia and caregivers to use technology. | 87.74% agree or strongly agree | - It is crucial to guarantee people with dementia and their caregivers are informed and aware of the existing technology and it's benefits while the access to it should be easy. […] (25843659) - It is important to ensure that the technology is usable, useful and the person knows what it is for and how to use it. (26196453) - Education and training (healthcare professionals and patients/caregivers), […] (25668793) - Information, training, research & development (25843659) - Education to those who will be dealing with people who have dementia (25283800) - […], knowing which technology to choose (25547050) - […] train staff, carers, family, people with dementia in use of technologies. (25327042) - More education & awareness needed (26193665) - […] lack of knowledge, […] (26067329) - Being aware of and being able to access it. […] (26055432) - […] if families don’t know about developments, they can’t integrate them… and everyone is time poor… (26099123) - On the one hand; implementation etc should not hinder innovation. A lot of innovation and research might not immediately be clear to health care professionals, patients and caregivers, but might turn out highly helpful. Second challenge: keeping this accessible to all! (25447271) - […] I think one challenges is how to make the technologies accessible, many of people with dementia and caregivers have less information on it, how to introduce the technology in community is a point to address. There are various assistive technologies in the market, if we would like to promote the use of it, will there be any conflicts? […] (25755060) - Time and care needed to make sure they understand (25283800) - Accessibility- not all people who are starting on there journey knows where to start accessing information- this can be very challenging- -and when they do it can be challenging & complicated. […] (26193665) - People with dementia might not know to use technologies or it might be challengeable to use. (25094761) - using technology not sure what is right and wrong […] (26055869) - […] Again training & education is possibly one of the biggest challenges i would see for caregivers and families to improve a parson quality of care who is living with dementia (26193665) - […] Ensuring people are aware if technology changes, ensuring that technologies work together if needed (i.e. things communicate with each other, or work together). Reduce duplicate labour (I don't want to have to type my details out 20 different times to use a single function, like a smart kettle). (25327554) - Reminders of how to use it and some person that can be contacted if there is a problem (25361561) - Fear of the unknown. Unknown skills to use but perhaps more so, […] (26071806) - 3. How to choose the right and necessary technologies addressing their needs could be a challenge (25571274) - Keeping carers and support groups up to date with developments […] (25500731) - […] In addition I think another challenge might be that there will be an abundance of technology available and the target population will find it difficult to decide for which one to go and what are the differences among the offer. (25942808) - Not one answer possible! Closely collaborating with different stakeholders; discussion about pros and cons; making a guide/ database with effective tools and anticipated type of benefits so that people can make a well informed choice (26109408) - In some societies, especially older people, do not use to use technological devices. They do not trust technologies, and try to avoid them as much as they can. If some of those people develop dementia, they probably would not prefer to use technological tools. Therefore, I believe there is a need for more training about technological devices in those minority groups. Older people are generally dependent on younger people for technological matters. Considering that the number of older people living alone is increasing day by day, it can be seen how much technological knowledge is needed in the future. Since dementia makes it difficult to use technological knowledge, these devices should be developed as user-friendly as possible. (24951005) | **New aspects**   - Also train staff - lack of information being passed on to those affected and carers - Provide information, education and training to use technology - It must be remembered that caregivers have little time when offering such training and information.   Lack of information and knowledge is a barrier for people living with dementia and caregivers to use technology.  **Agreed amendment:**  It is essential to provide education for people living with dementia and caregivers and training care staff about how to use the technology. A lack of information and knowledge is a barrier to use technology. |
| Technology does not always fit well in the current healthcare practices and this is a barrier to its use. | 74.53% agree or strongly agree |  | **Team discussions and PPI input:**   - Relevance to Living with dementia - What does this mean?   No supporting comments identified. Exclude. Consensus not reached. |
| Care professionals should support people with dementia and caregivers to learn how to use technology if they need it. | 83.02% agree or strongly agree | - People with dementia may have issues learning new skills and adapting to technology. They need a lot of support to set it up and someone to monitor. Family members who are older may not have skills to assist. Some may not have WiFi. (26059552) - It’s really challenging to teach people with Alzheimers or dementia how to use technology. My aumt can’t even use her phone now. You would really want to ensure that technology is the right tool before investing loads of money in it. Zoom for families is something we would like to be able to use with our relative who has Alzheimers. (26162728) - The medical team in GP surgeries need to be more patient as not all PLWD and there care partners are able to make appointments using technologies such as they ask us to use (26114813) - Have special qualified people in these areas available to help carers and people with dementia on a one to one basis..(25337402) - Simple technologies that the carer is trained and supported to use for the person with dementia may be appropriate. Keep in mind the carers rarely have time to learn and use these. (25330345) - I thunk having someone come to the house and help install and advise what could help and maybe come back a month later and see if the technology was even used and if bot ask why and what needs to be changed with the tech to help more. (25326789) - Updates, contact, practical assistance, support meetings (26065387) - […] extensive support/easy to reach helpdesks (26031716) - Grants, or hiring equipment may help more people avail of it. Easy to use instructions for the instructor. Possible online back up? (26054921) - Getting folk to adopt the technology as soon as possible (25279026) - Technology can be complicated to learn and impossible for the person with dementia. I have been offered various "assistive devices" which create a lot of stress and time to learn and the person I care for cannot learn to use them. (25330345) - […] no support to install the technology and train the person with dementia or their caregiver, the technology may support the person with dementia but it may also add to the workload of the carer, many assistive tech projects I have been involved in (especially if coordinated by a technical partner rather than a clinical partner) place an emphasis on new and innovative tech solutions whereas the PPI group/end users want something simplier. Improving the user experience is not what the tech partners are interested in doing - they want to explore the new tech, push boundaries etc. and they is the way their funding has been set up. As long as this is the main emphasis, people with dementia and their caregivers will not get what they need in the way that they need it and this is a huge challenge/barrier to the use of supportive technologies. | **New aspects**   - people living with dementia and caregivers often require extensive, easily accessible support with the set-up and training to help them use the technology. - Older family members may not have the technical skills to help and may lack basic resources such as Wi-Fi.   **Agreed amendment:**  It is important that experts are available to provide individual, personalised support during installation and continued use of the technology (e.g. dedicated end-user support and providing training through community services). |
| Researchers and developers should plan how technology will be set up and used, for example who will install it and who will teach people with dementia how to use it. | 86.79% agree or strongly agree | - More focus on implementation and actual uptake (25547050) - Implementation […] for technologies should also be considered from day one, (26056147) - Think from the beginning on who will be the one implementing it in practice: care professionals? informal caregivers? technology developers? […] (24957622) - […] Focus on implementation. (25942471) - Invest time and funding in practical efforts to get technology implemented (e.g. time for caregivers to receive technology coaching, technology coaches that can help people with dementia, etc.) (25547050) - […] And make sure that the implementation of technologies into people's lives (for example also discussing the options with the public health sector and insurances) is equally important (25942808) - It is important to understand how policies around dementia care or social policies support the implementation/adaptation of technology in dementia care. If they has to be obsorbed into the care ecosystem, then who is going to pay for it? government or the people.. If government then how is that money being raised(general taxes, earmarked taxes, co-payment??) or if by the people, then it needs to be heavily subsidised to avoid high out of pocket payments and financial burden( especially if the family has more than one person with dementia) (26084377) - Implementing technologies sustainably into the lives of people with dementia; creating evidence while technologies advance. (25383836) - See my previous answer about multi-disciplinary collaborations in research. I also believe that there should be more focus on 1) post-implementation research into (cost-)effectiveness of technologies 2) longitudinal research with technologies to see how their use, usability and effectiveness change over the dementia journey 3) comparitive research, comparing different technologies or combinations of technologies to each other. (24952169) - 1. Accuracy of technology 2. Implementation of technology 3. STandardisation of products (too many products for the same purpose) 4. Integration of products in a rather tense market (24951560) - Technologies are developed so quick that research can’t keep up with the new developments. So challenges will be that technologies will be developed that are not evaluated in a research context or without the needs of people living with dementia in mind (24957622) - as before - it depends what your question is about. You seem to be locating issues onto technology but this is only one part of the problem. We need to learn from the history of success and failure in co-creating and implementing technology into the lives of people with dementia and other long term conditions. (26046796) - Do better research, and follow scientific development cycles of innovation. (25447271) | **New aspects**   - From the outset, the focus should be on who will later implement the technology in practice. This ensures a practical approach. - Developers often focus on innovative solutions while the actual needs of end users are neglected. They often do not consider how technologies can be integrated into actual routines. It is also frustrating when technologies are not compatible with each other. Sustainable implementation in everyday life is therefore a challenge.   Researchers and developers should consider the implementation at an early stage.  Researchers and developers should plan how technology will be setup and used, including who will install it and teach people with dementia and their caregivers.  The focus should be on those who will use technology in everyday environments to ensure it meets user needs, integrates into routines and is compatible.  **Agreed amendment:**  Researchers and developers should focus on who will use technology in everyday environments to ensure it meets user needs, integrates into routines and is compatible. |
| New statement(s) | NA |  | NA |
| **Ethical or fair use of technology** | | | |
| Technologies need to be developed to protect and maintain the privacy of the person with dementia. | 89.62% agree or strongly agree | - […]. • Technologies must be designed with strong data security measures and give users control over what information is shared and when […] (25861755) - Finger print/facial recognition to ensure only they can access it with trusted carers support. (26184160) - I don't have solutions to all of these, though maybe there needs to be more recognition of how people transition from being IT-iindependent to being helped by someone else, e.g. that there is a recognition that password sharing may be OK. (26196453)   **Future Challenge: Ethical considerations**   - Balancing autonomy with safety. […] (25327554) - […] privacy (26067329) - Privacy! (26057431) - Ethical and privacy issues (25952113) - privacy and personal data security, […] (25977236) - Ethical dilemma- that who will have access to this data. For eg: night time sensors, or movement trackers like GPS. It is important to understand who will have access to this data as it has serious implications on autonomy of a person. […] (26084377) - […] Technologies such as tracking devices or monitoring systems, require access to personal and health data raising concerns around privacy and autonomy of people with dementia. […] (25861755) - I see the protection of privacy being a key issue (25942520) - [...] privacy and data protection. (26162728) - Privacy will be an important issue to address, especially when commercial partners are involved. […] (25937856) - […] not making people with dementia too dependent on tools and undermining their autonomy prematurely (as a person with dementia quoted at the recent Alzheimer Europe conference possibility of integrating different tools / difficulty of using many different tools simultaneously freedom of choice for different applications versus care organisations choosing often for one type of video care how to choose between so many different tools? (26109408) - I worry about how people will move from having an independent online life (email, banking, social media etc) to being reliant on others to help them. How does this happen without for example sharing passwords? Also, as life becomes increasingly reliant on technology, e.g. you need a smartphone for buying tickets, verifying your identity, etc, or you need a computer to do your shopping, it seems that people with cogntiive impairment may become more disabled earlier than used to be the case in simpler times, e.g. when you paid in cash for everything. […] (26196453) - […] Another important factor is owning of the product. If the technologies like robo pets/ companions is being used, studies show that they develop attachment with those robots pets, and if there is any technical glitch or if the pet has to be replaced due to some technical issues, the persons with dementia, could not take it as they are emotionally attached to it and it may cause severe depression. (26084377) - […] Third, the vulnerability of people with dementia to online and other scams is a public health problem. (26196453) - Dementia patients are most at risk to scamming and hacking because they may not see the risk in sharing information (26184160) | **New aspects**   - Technologies such as monitoring and tracking devices require access to personal and health data, which raises concerns about the privacy and autonomy of people living with dementia. This applies in particular when commercial partners are given access to personal data. - The vulnerability of people living with dementia to online fraud is seen as a growing problem.   **Team discussions and PPI input:**  Ownership of technological products, such as robotic companions, can also have a significant emotional impact on people living with dementia.  Technologies must protect against fraud and maintain the privacy of people living with dementia.  **Agreed amendment:**  Technologies for people living with dementia must protect privacy and autonomy and acknowledge their vulnerability to data sharing and fraud. |
| Technologies should be designed to help people with dementia make their own choices while also keeping them safe. | 91.51% agree or strongly agree | - […] Need to consider the ethical questions - e.g. the person with dementia may consent to using a device but in the moment, they should be able to take off or stop the device if it is bothering them. There was a presentation at the recent AE conference where a bracelet couldn't be removed from the person's arm. This is NOT ideal. (25517807) - ethical use of technologies and 'best interest' decisions being made that are in the best interest of the families and professionals and not necessarily of the person living with dementia. (26090971) - Focus on health and safety (25475638) | **New aspects**   - The autonomy of people living with dementia must be respected. - They should be able to refuse or remove technologies such as devices if they become uncomfortable. - decisions are often made in the ‘best interests’ of families and professionals, but this does not necessarily correspond to the needs of the person living with dementia.   **Agreed amendment:**  Technology should support the autonomy of people living with dementia, allowing them to make decisions on what is comfortable for them while ensuing safety. |
| Care professionals and researchers shouldn't just talk about the fair use of technology; they should apply this to their practice. | 83.96% agree or strongly agree | - […], equitable access to all patients and carers (support by state policies) (25668793) - consideration of wider socio-economic, cultural and income level settings (25092956) - […] Technologies may also drive inequity, especially if not covered by public care funding and if not designed on the basis of universal design principles. (24952169) - They could increase the gap in access to support between rich and poor countries and communities (25942679) - Equitable access […] (25668793) - Equity in acces (25092956) - […] inequity due to the costs inequity due to different levels of education/tech savyness (26031716) - equity of (digtial care), accessibiity of digital tools for people of different cultural backgrounds and literacy. […] (26109408) | **New aspects**   - equal access to technology must be guaranteed - different socio-economic and cultural backgrounds must be taken into account - technologies can worsen inequalities if they are not covered by public healthcare - reuse “old” technology   **Agreed amendment:**  Care professionals and researchers should ensure equal access to technology, taking socioeconomic and cultural factors into account and improve accessibility. |
| Researchers and developers should follow standards and guidelines about the ethical and fair use of technology to support people with dementia. | 92.45% agree or strongly agree | - Rules and regulations for good and appropriate technology (for manufacturers) (24951560) - Use strict GDPR rules, not lowest common denominator eg US. (26057431) - Use of AI with the necessary legislation and regulation. (24961604) | **New aspects**   - the use of AI can also lead to challenges in the future if its use is not regulated.   **Agreed amendment:**  Technology in dementia care must meet best practices for ethical standards and regulations for fair use as well as artificial intelligence. Researchers and developers must ensure this happens during design and development. |
| New statement(s) | NA |  | NA |

**Other aspects not suitable for statements but maybe for results**

Important features can include large screens, the ability to receive messages, make calls and stay in touch with others. Reminders for tasks or appointments are helpful. Other interesting features are the security and monitoring of people living with dementia, for example through GPS tracking.

Incorporate exercises or functions that support cognitive function into the technology and focus on health and safety when designing technologies.

- Large screen, create lists, tasks, news, what day is it, who's calling etc Ability for family to add to list/news from afar. (25396757)
- If possible, GPS tracking with people with dementia is important. Wearables with GPS tracking can provide peace of mind for caregivers by alerting them if the person with dementia wanders outside a predefined safe zone. […] (25094761)
- […] 2. Use friendly language. […] (25571274)
- Build in cognitive function in everyday technology (25363444)
- Bigger buttons. Single word to Inform dementia patients (25026586)
- […] text size use dementia friendly colours on screens use of plain English avoid computer jargon which means nothing to me expense in obtaining technology (26055869)
